# Controlling Cytokine Storm Is Vital in COVID-19

**DOI:** 10.3389/fimmu.2020.570993

**Published:** 2020-11-30

**Authors:** Lu Tang, Zhinan Yin, Yu Hu, Heng Mei

**Affiliations:** ^1^Institute of Hematology, Union Hospital, Tongji Medical College, Huazhong University of Science and Technology, Wuhan, China; ^2^Hubei Clinical Medical Center of Cell Therapy for Neoplastic Disease, Wuhan, China; ^3^Zhuhai Institute of Translational Medicine, Zhuhai People’s Hospital Affiliated with Jinan University, Jinan University, Zhuhai, China; ^4^The Biomedical Translational Research Institute, Faculty of Medical Science, Jinan University, Guangzhou, China

**Keywords:** COVID-19, cytokine storm, ACE2, IL-6, immunoregulatory therapy

## Abstract

Corona virus disease 2019 (COVID-19) has caused a global outbreak and severely posed threat to people’s health and social stability. Mounting evidence suggests that immunopathological changes, including diminished lymphocytes and elevated cytokines, are important drivers of disease progression and death in coronavirus infections. Cytokine storm not only limits further spread of virus in the body but also induces secondary tissue damage through the secretion of large amounts of active mediators and inflammatory factors. It has been determined that cytokine storm is a major cause of deaths in COVID-19; therefore, in order to reverse the deterioration of severe and critically ill patients from this disease, the cytokine storm has become a key therapeutic target. Although specific mechanisms of the occurrences of cytokine storms in COVID-19 have not been fully illuminated, hyper-activated innate immune responses, and dysregulation of ACE2 (angiotensin converting enzyme 2) expression and its downstream pathways might provide possibilities. Tailored immunoregulatory therapies have been applied to counteract cytokine storms, such as inhibition of cytokines, corticosteroids, blood purification therapy, and mesenchymal stem cell therapy. This review will summarize advances in the research of cytokine storms induced by COVID-19, as well as potential intervention strategies to control cytokine storms.

## Introduction

Pathogenic human coronavirus infections, such as severe acute respiratory syndrome (SARS) and Middle East respiratory syndrome (MERS), could cause fatal lower respiratory tract infections and extra-pulmonary manifestations (1–3). The Coronavirus Disease 2019 (COVID-19), first reported in Wuhan, China, has raised acute and grave global concern since December 2019 (4, 5). On March 11, 2020, the world health organization (WHO) officially declared a global pandemic status for COVID-19, which is a great threat to people’s health and social stability. In the early stages of COVID-19, severe acute respiratory infection symptoms occurred, and some patients rapidly developed acute respiratory distress syndrome (ARDS), acute respiratory failure, and other serious complications (6, 7). There is considerable evidence to show that immunopathological changes, including diminished lymphocytes and elevated cytokines, are important drivers of disease progression and death of COVID-19 patients, especially those who are critically ill (8). Early detection and control of cytokine storms may effectively prevent disease progression and reduce the mortality rate. This review will summarize research progress on cytokine storms occurring in COVID-19 as well as potential intervention strategies.

### Cytokine Storm and Cytokine Release Syndrome

The cytokine storm, previously reported in rheumatoid arthritis and graft-versus-host disease (GVHD), has been used to describe the overactive immune responses that can be triggered by a variety of factors, such as virus infection, autoimmune disease, and immunotherapies (8–10). Under the normal physiological state, the levels of pro-inflammatory cytokines and anti-inflammatory cytokines in the body are kept in relative balance, which can be broken by abnormal activation of a variety of immune cells (such as dendritic cells, macrophages, and lymphocytes) during viral infections. These abnormally activated immune cells could release large amounts of cytokines, among which the pro-inflammatory cytokines could promote more immune cells in a positive feedback loop. The formation of cytokine storm leads to “suicide attack” that not only contributes the elimination of pathogenic microorganisms but also causes tissue toxicities affecting a wide variety of organs (**Table 1**) (9). The cytokine release syndrome (CRS), a kind of systemic inflammation syndrome caused by cytokine storm, was previously observed in patients infected with SARS-CoV and MERS-CoV, as well as in leukemia patients receiving engineered T cell therapy (8, 9, 11, 12). Mild cases are characterized by fever, fatigue, headache, rash, arthralgia, and myalgia. Patients with more severe symptoms usually present with high fever, headache, fatigue, diffuse intravascular coagulation (DIC), shock, multiple organ failure (MOF), or even death (9, 11). Common laboratory abnormalities include cytopenia, elevated creatinine and liver enzymes, high levels of C-reactive protein (CRP), and deranged coagulation parameters (9, 11). Lee et al. reported a modified grading system for the severity of CRS regardless of the inciting agent, which defined mild, moderate, severe, life-threatening symptoms, and even death. This grading system is also used to guide clinical decisions in CRS (11). Vigilant supportive care is recommended for every grade; immunosuppression should be used in all patients with grade 3 or 4 CRS and instituted earlier in patients with extensive comorbidities or the elderly (11).

**Table 1:** Clinical signs and laboratory findings about cytokine storms.

Organ	Clinical signs and laboratory findings
Constitutional	Fever, rigors, headache, malaise, fatigue, anorexia, myalgias, nausea, vomiting
Pulmonary	Tachypnea, hypoxemia
Hematologic	Anemia, thrombocytopenia, neutropenia, febrile neutropenia, lymphopenia, B-cell aplasia, hypofibrinogenemia, bleeding, elevated D-dimer, prolonged prothrombin time, prolonged activated partial prothrombin time, disseminated intravascular coagulation
Gastrointestinal	Nausea, diarrhea, emesis
Cardiovascular	Tachycardia, widened pulse pressure, hypotension, arrhythmias, QT prolongation, increased cardiac output (early), potentially diminished cardiac output (late)
Renal	Acute kidney injury, hyponatremia, hypokalemia, hypophosphatemia, tumor lysis syndrome, azotemia
Hepatic	Transaminitis, hyperbilirubinemia
Neurologic	Headache, mental status changes, confusion, delirium, word finding difficulty or frank aphasia, hallucinations, tremor, dymetria, altered gait, seizures
Skin	Rash, edema

### Cytokine Storm Induced in Viral Infection

The virus can promote the activation of immune cells (such as T cells, B cells, macrophages, dendritic cells, neutrophils, monocytes) and resident tissue cells, resulting in the production of large amounts of inflammatory cytokines (13, 14). During the flu virus infection, innate immune responses get started through the cascade amplification reactions of interferon stimulated gene expression, and type | interferon (IFN) is mainly produced by monocytes, macrophages and dendritic cells (15). Serum levels of interleukin 8 (IL-8), IP-10 (interferon-induced protein 10), MCP-1 (monocyte chemoattractant protein-1), MIP-1 (macrophage inflammatory protein-1), MIG (monokine induced by IFN-γ) and CXCL-9 (CXC chemokine ligand-9) were abnormally elevated in H5N1 influenza virus infection, while IL-8, IL-9, IL-17, IL-6, IL-15, TNF-α (tumor necrosis factor-α), IL-10 were increased in H1N1 influenza virus infection (16–18). Earlier researches demonstrated that serum levels of proinflammatory factors IFN-γ, IL1β, IL-6, IL-12, IL-18, IP-10, MCP-1, and CCL2 (CC chemokine ligand-2), CXCL-10 and IL-8 are positively correlated with lung inflammation and extensive lung tissue injury in SARS patients (19–21). Whereas, the levels of serum pro-inflammatory cytokines IL-6, IFN-γ, TNF-α, IL-15, IL-17, and chemokines IL-8, CXCL-10, and CCL5 were significantly increased in severe MERS patients (22, 23). Among numerous molecules that increase in virally-mediated cytokine storms, IL-6, IFN-γ, IL-1β, IL-8, IL-10, and TNF-α are of crucial importance (9, 24, 25). The occurrence of cytokine storm has been reported to be one of the main causes of death in patients with SARS-CoV, MERS-CoV, and influenza virus infections (8, 26). Similarly, cytokine storm is also a common feature of severe cases in COVID-19, and elevated levels of serum IL-6 and CRP correlate with respiratory failure, ARDS, MOF and adverse clinical outcomes (27, 28).

### Pathophysiology of Cytokine Storm

**Figure 1:**
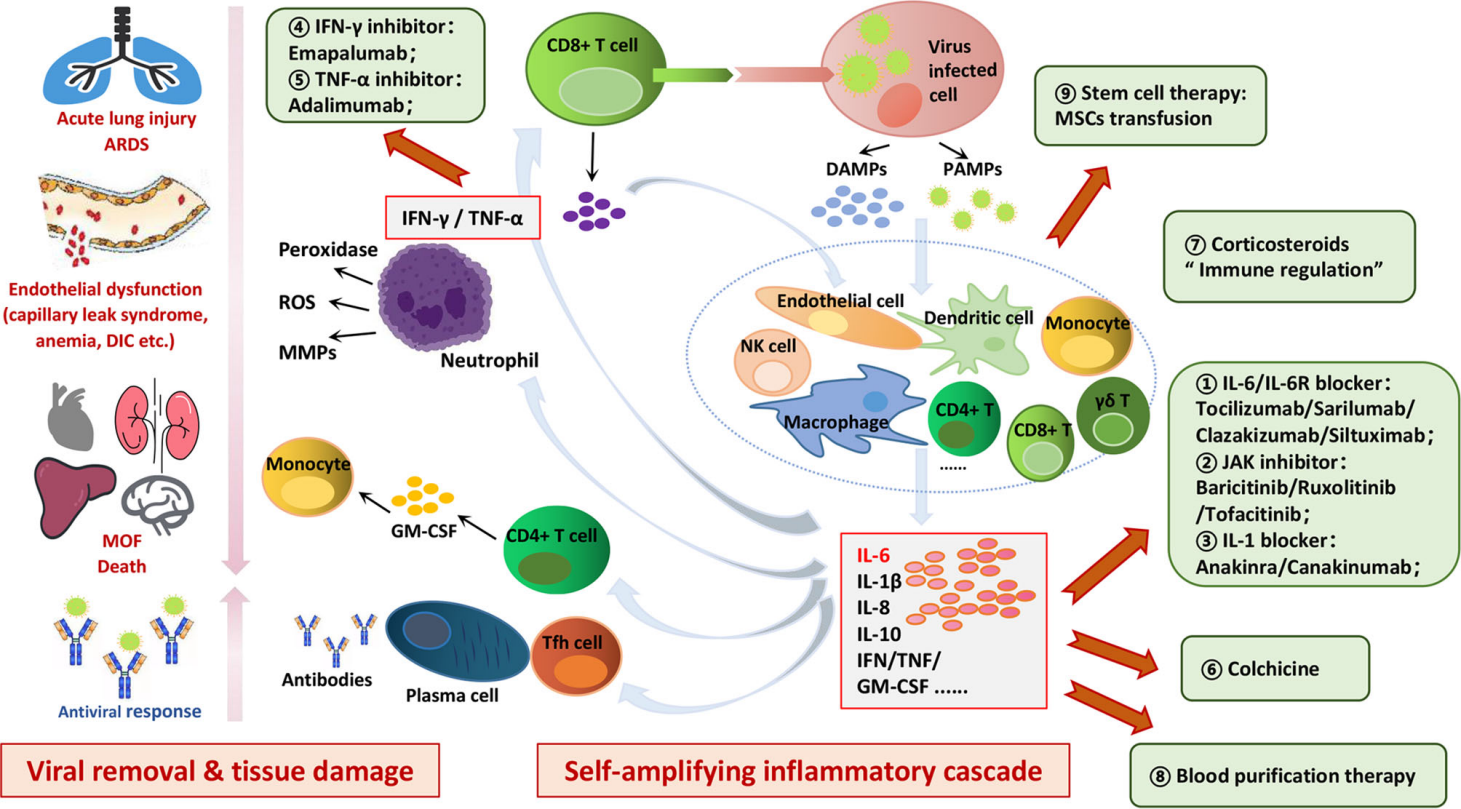
Mechanisms and hazards of cytokine storms induced in COVID-19 and potential therapeutic targets. Viral infection can induce antiviral responses in neighboring cells as well as recruit innate and adaptive immune cells, such as macrophages, dendritic cells, T cells, B cells and NK cells, leading to self-amplifying inflammatory cascade in a positive feedback loop manner. Cytokine storm not only limits further spread of virus in the body but also induces secondary tissue damage through the secretion of a large number of active mediators and inflammatory factors. The successive occurrences of acute lung injury, abnormal alterations in vascular hemostasis, and cytokine-mediated tissue damage can eventually result in MOF. Potential therapeutic targets to control cytokines storms in COVID-19 are as follows: IL-6/IL-6R blocker; JAK inhibitor; IL-1 blocker; IFN-γ inhibitor; TNF-α inhibitor; colchicine; corticosteroids; blood purification therapy; stem cell therapy.

Both damage-associated molecular patterns (DAMPs) and pathogen-associated molecular patterns (PAMPs) are produced upon viral infection, which can activate antiviral responses in neighboring cells as well as recruit innate and adaptive immune cells, such as macrophages, natural killer (NK) cells, and gamma-delta T (γδ T) cells (**Figure 1**) (24, 29–33). Downstream production of interferons promotes intracellular antiviral defenses in neighboring epithelial cells which may limit viral dissemination, while the release of IL-6 and IL-1β from other immune cells promotes recruitment of neutrophils and T cells (29). Subsequently, the activation of T cells or lysis of immune cells induces secretion of IFN-γ and TNF-α, leading to the activation of immune cells and endothelial cells with further release of pro-inflammatory cytokines in a positive feedback loop manner (9). Although these inflammatory cytokines promote T follicular helper (Tfh) cell differentiation, B cell germinal center formation and antibody production, as well as Th1 (T helper 1) cell differentiation and cytotoxic CD8+ T cell generation to help viral removal, tissue damage caused by them cannot be ignored (34, 35). Activated neutrophils release leukotrienes and reactive oxygen species (ROS) that induce local pneumocyte and endothelial injury, directly leading to acute lung injury (29). Inflammatory mediators promote neutrophil release of nuclear deoxyribonucleic acid (DNA) to form neutrophil extracellular traps (NETs) which can snare pathogens as well as contribute to thrombi formation (33). This process, termed immuno-thrombosis, can also amplify the production of cytokines and is exemplified by links of thrombin with inflammasome activation and production of IL-1 (36). As vascular endothelial cells would be exposed to circulating cytokines and other immune mediators, coagulation disorders (such as capillary leak syndrome, thrombus formation, and even DIC) can also be caused by endothelial cell dysfunction in cytokine storms, indicating the crosstalk between hemostasis and cytokines (24, 33). High levels of circulating inflammatory cytokines can cause cell death as well as tissue damage, whereas their promotion of macrophages activation can lead to erythro-phagocytosis and anemia (24, 33). The successive occurrences of acute lung injury, abnormal alterations in vascular hemostasis, and cytokine-mediated tissue damage can eventually result in MOF (24, 33, 37).

As discussed above, cytokine storm not only limits further spread of virus but also induces secondary tissue damage through the secretion of large amounts of active mediators and inflammatory factors (**Figure 1**) (24, 29–33, 35, 38, 39). Thus, it can be seen that inhibition of this self-amplifying inflammatory cascade may not only control tissue damage, but also impair viral clearance. Unlike that in viral infection, the occurrence of cytokine storm in CAR-T therapy is secondary to T cell-mediated killing of tumor cells, and inhibition of this self-amplifying inflammatory cascade has no influence on therapeutic efficacy (9, 29). Nevertheless, it has been proved that antiviral immune responses and tissue toxicity could be at least partially uncoupled; therefore, inhibition of select arms of innate immune responses could limit tissue toxicity while not greatly suppressing ongoing antiviral immunity (29).

### Clinical, Pathological, and Serological Manifestations in COVID-19

The incubation period of COVID-19 patients ranged from 1 to 14 days, and mostly from 3 to 7 days (8, 40). Common symptoms at onset of illness were fever, dry cough, myalgia, fatigue, dyspnea, and anorexia; however, a few patients presented initially with atypical symptoms, such as diarrhea and nausea (6, 40). Most patients had good results with treatment, while the elderly patients and those with chronic diseases usually had poor prognoses. Although most patients with COVID-19 had mild and moderate symptoms, severe and critically ill patients progressed rapidly to acute respiratory failure, ARDS, metabolic acidosis, coagulopathy, septic shock, and MOF (7, 40–42). The autopsy report in the journal The Lancet Respiratory Medicine showed typical ARDS-like lung injury with lymphocyte infiltration, liver injury with moderate micro-vesicular steatosis, mild lobular and portal activity (43). COVID-19 patients usually revealed significantly reduced lymphocyte counts and increased inflammatory factors, especially those with severe illnesses (6, 40, 44). Wang et al. reported that the neutrophil count and D-dimer continued to increase in non-survivors, whereas lymphocyte counts continued to decrease until death occurred (40). Neutrophilia may be related to cytokine storms induced by virus invasion, and coagulation activation may be related to sustained inflammatory responses (40).

### Cytokine Storm Is a Key Determiner in the Fate of COVID-19

Huang et al. observed that patients in intensive care unit (ICU) showed higher levels of plasma inflammatory cytokines IL-2, IL-7, IL-10, G-CSF (granulocyte colony-stimulating factor), IFN-γ and MCP and TNF-α than non-ICU patients (6), indicating the positive correlation between the cytokine storm and the severity of illness. These cytokines suggested not only Th1 responses but also Th2 responses in COVID-19, which differed from SARS-CoV infections (6). After being infected with the SARS-CoV-2, CD4+ T cells were activated and differentiated into Th1 cells to secrete pro-inflammatory cytokines, such as IL-6, IFN-γ, and GM-CSF (granulocyte-macrophage colony stimulating factor) (6, 45). GM-CSF could activate mononuclear cells to promote further release of IL-6 and other pro-inflammatory cytokines, leading to the generation of cytokine storms (45). Therefore, IL-6 and GM-CSF released by T lymphocytes and mononuclear cells may be the key link of cytokine storm in COVID-19 (45). Moreover, the activation of monocytes may suggest that the cytokine storm in COVID-19 is closely related to the destruction of the balance between innate and adaptive immunity.

Recent studies also showed that the level of IL-6 in severe cases was significantly higher than that in mild and moderate cases, but the levels of CD4+ T cells, CD8+ T cells and NK cells were decreased, indicating the immunosuppression in severe COVID-19 patients (44). Although peripheral CD4+ T cells and CD8+ T cells were significantly reduced, the number of Th17 cells increased and CD8+ T cells were highly cytotoxic, which further suggested that cytokine storms may aggravate tissue damage (43). Meanwhile, T lymphocyte cells were excessively activated during the cytokine storm in COVID-19 patients, which may be accompanied by severe immune dysfunctions (43). The cytokine storm can directly damage the pulmonary capillary mucosa, promote alveolar edema, and further induce the diffusion of inflammatory cytokines, thus resulting in the damage of alveolar structure and pulmonary ventilation dysfunction (25, 46). In addition, the cytokine storm is associated with the sequence and severity of organ dysfunction in multiple organ dysfunction syndrome (MODS) (37). Therefore, the cytokine storm may be an important factor that affects the fate of patients with COVID-19 pneumonia.

## Possible Mechanisms of Cytokine Storm in COVID-19

### Hyperactivated Innate Immune Responses

During the process of antiviral immune responses, innate and adaptive immune responses interact with each other and cooperate closely to produce immune protection (47). There is a time limit for adaptive immune responses, which usually gets initiated 4 to 7 days after infection. Unlike adaptive immune responses, innate immune responses occur immediately after infection and are fully involved in virus clearance. However, the innate immunity is relatively weak in virus clearance, and adaptive immunity is the key factor in complete elimination of the virus (47). If the body does not generate effective adaptive antiviral responses in time to clear the virus, the innate immune responses will be strengthened, which cannot eliminate the virus effectively and even lead to systematic inflammation responses with uncontrolled release of inflammatory cytokines (48, 49). The latest studies have shown that the average age of severe and critically ill patients is higher than that of mild cases (66-years old vs. 51-years old), and severe cases are more likely to have other chronic diseases (72.2% vs. 37.3%) (40, 50). As for the elderly patients and those with chronic diseases, it takes a longer period of time to generate effective adaptive immune responses due to the deterioration of immune functions. These patients only rely on strengthening the innate antiviral immune responses in the early stages of infection, leading to a higher risk of cytokine storms, earlier onset of severe illness, and a higher mortality rate. Although the kinetics of the responses to SARS-CoV-2 fit with models of the induction of conventional antiviral immunity, it is still unclear whether immune hyperactivity is due to ongoing viral replication or immune dysregulation (33). Pyroptosis is a highly inflammatory and caspase-1-dependent form of programmed cell death that occurs most frequently upon infection with intracellular pathogens and is a part of the antimicrobial responses, which may also play a role in COVID-19 pathogenesis (51, 52). Rapid viral replication that causes increased pyroptosis may lead to a massive release of inflammatory mediators (51). Taken together, viral escape to avoid anti-viral immunity, together with genetic or acquired defects in host defense, may impair viral clearance, resulting in inappropriate immune activation and consequently causing cytokine storms (52). In a word, the exaggerated activation of innate immunity may be an important factor in the formation of cytokine storms in COVID-19 (29).

### Dysregulation of ACE2 and Its Downstream Pathway

Angiotensin converting enzyme 2 (ACE2) primarily catalyzes the breakdown of angiotensin II (AngII) to maintain the homeostasis of renin-angiotensin-aldosterone system (RAAS) as a pivotal counter-regulator, which is crucial for the physiology and pathology of most human organs (53, 54). Significant to COVID-19, ACE2 has been established as the functional host receptor for SARS-CoV-2, and many factors have proved to be associated with both altered ACE2 expression and disease severity and progression, including age, sex, ethnicity, medication, and several co-morbidities (such as cardiovascular disease, metabolic syndrome, and lung cancer) (53–57). Recent studies have also revealed potential roles of ACE2 in regulating immune responses rather than merely being a viral-binding receptor in COVID-19 (53–55, 58–61). Chen’s team found the expression axis “mir-125b-5p-ACE2-IL-6” existed in lung adenocarcinoma, in which mir-125b-5p inhibited the expression of IL-6 through promoting the up-regulation of ACE2 (58). Once bound by SARS-CoV-2, the ACE2 expression (including mRNA level and enzyme activity) on the surface of host cells were significantly decreased, and then IL-6 in the toll-like receptor signaling pathway might influence the immune system as the downstream effector (53, 54, 58). Therefore, the dysregulation of ACE2 induced by SARS-CoV-2 infection may further cause cytokine storms and pneumonia, and targeting to the upstream regulator mir-125b-5p may provide a new way for the control of COVID-19 (58, 60). More interestingly, the spike protein of SARS-CoV has been previously demonstrated to downregulate ACE2 expression, thus resulting in over-production of AngII (the downstream of ACE2) by the related enzyme ACE (62, 63). Similarly, it could be hypothesized that SARS-CoV-2 may downregulate ACE2 receptors and thereby leading to an over-production of AngII, which may be another possible explanation of cytokine storm in COVID-19 (59, 61). ACE2 molecules on the cell surface are occupied by SARS-CoV-2, and then AngII increases due to a reduction of ACE2-mediated degradation (59, 61). SARS-CoV-2 itself activates nuclear factor kappa B (NF-κB) *via* pattern recognition receptors (PPRs), and the accumulated AngII in turn induces the activation of the IL-6 amplifier through enhanced activation of NF-κB pathway and IL-6-STAT3 (signal transducers and activators of transcription family 3) axis (61). IL-6 is a key factor in this positive feedback loop, ultimately leading to the release of cytokines out of control (64). More importantly, IL-6 is a major functional marker of cellular senescence, and the age-dependent enhancement of the IL-6 amplifier may correspond to the age-dependent increase of COVID-19 mortality (61, 64).

## Controlling Cytokine Storm is Vital in COVID-19

Lessons should be learned from the outbreak of SARS-CoV and MERS-CoV to accumulate valuable experience and insights on how to effectively treat COVID-19 pneumonia patients. There are three progressive stages upon SARS-CoV-2 infection: early infection, pulmonary phase, and hyper-inflammation phase (65). Targeted treatments are urgently needed to prevent the occurrence of cytokine storms, and the early infection stage with no or mild symptoms is the key period for active treatment to control further deterioration (65). Antiviral drugs that inhibit virus transmission and destroy virus replication can reduce direct cell damage caused by COVID-19, and appropriate combinations with immunoregulatory therapies that inhibit hyper-activated inflammatory responses can resist cytokine storms triggered by the virus (**Figure 1** and **Table 2**) (72, 73). Multiple clinical trials have been initiated to investigate potential interventions to control cytokine storms in patients with COVID-19, mainly including direct inhibition of cytokines and immunomodulatory therapies (**Figure 1** and **Table 2**).

**Table 2:** Ongoing clinical trials of strategies to control cytokine storms in COVID-19.

Trial identifier	Participants	Trial design	Therapy	Mechanism	Adverse effects
ChiCTR2000029765	Adult (18–85 years), moderate/severe/critically ill patients	A multicenter, randomized controlled trial	Tocilizumab vs. Standard medical care	IL-6 receptor blocker	Infections; skin rash; anemia; neutropenia; lymphopenia; liver enzyme elevations etc.
ChiCTR2000030796	Diagnosed cases	A retrospective study	Tocilizumab		
ChiCTR2000030894	Adult (18 Years to 65 Years), moderate/severe patients	A multicenter, randomized, controlled trial	Tocilizumab combined with Favipiravir vs. Standard medical care		
NCT04332913	Adult, severe/critically ill patients	A prospective, observational study	Tocilizumab		
NCT04322773	Adult, severe/critically ill patients	An open-Label, multicenter sequential, cluster randomized trial	Tocilizumab vs. Sarilumab vs. Standard medical care		
NCT04317092	Adult, severe/critically ill patients	A multicenter, single-arm, open-label, phase 2 study	Tocilizumab		
NCT04320615	Adult, severe/critically ill patients	A randomized, double-blind, placebo-controlled, multicenter, phase 3 trial	Tocilizumab vs. Placebo		
NCT04306705	Adult, severe/critically ill patients	A retrospective study	Tocilizumab vs. Standard medical care vs. Continuous renal replacement therapy		
NCT04315480	Adult, severe/critically ill patients	A phase 2 Simon’s optimal two-stages trial	Tocilizumab		
NCT04335071	Adult, severe/critically ill patients	A multicenter, double-blind, randomized controlled phase II trial	Tocilizumab vs. Placebo		
NCT04324073	Adult, moderate/severe/critically ill patients	A multiple, open-label, randomized controlled trial	Sarilumab vs. Standard medical care		
NCT04315298	Adult, severe/critically ill patients	An adaptive phase 2/3, randomized, double-blind, placebo-controlled Study	Sarilumab vs. Placebo		
NCT04327388	Adult, severe/critically ill patients	An adaptive phase 3, randomized, double-blind, placebo-controlled trial	Sarilumab vs. Placebo		
NCT04341870	Adult (18–80 years), moderate/severe/critically ill patients	a multicenter open-label 1:1 randomized controlled trial	Sarilumab vs. Azithromycin vs. Hydroxychloroquine		
NCT04348500	Adult patients with pulmonary involvement who have not yet required mechanical ventilation and/or ECMO	A single center, randomized, double-blind, placebo-controlled, exploratory phase II study	Clazakizumab vs. Placebo	IL- 6 monoclonal antibody	
NCT04322188 (66)	Adult, severe/critically ill patients	A single-center observational cohort study	Siltuximab vs. Standard medical care		
NCT04329650	Adult, severe/critically ill patients	A phase 2, randomized, open-label study	Siltuximab vs. methylprednisolone		
NCT04318366 (67)	Adult, moderate to severe patients	A retrospective cohort study	Anakinra vs. Standard medical care	IL-1 receptor antagonist blocking IL-1α and IL-1β	Infections; skin rash; anemia; neutropenia; lymphopenia; liver enzyme elevations etc.
NCT04330638	Adult, severe/critically ill patients	A prospective, randomized, factorial design, interventional Study	Anakinra vs. Tocilizumab vs. Siltuximab vs. Usual Care		
NCT04341584	Adult, severe/critically ill patients	A multiple randomized controlled trial	Anakinra vs. Standard medical care		
NCT04339712	Adult, severe/critically ill patients	A non-randomized, open-label trial	Anakinra vs. Tocilizumab		
NCT04365153 (68)	Adult, severe/critically ill patients with cardiac injury	A double-blind, randomized controlled trial	Canakinumab vs. Placebo	IL-1β monoclonal antibody	
NCT04348448 (69)	Adult (18–100 years), severe/critically ill patients	A prospective, observational study	Canakinumab		
ChiCTR2000030089	Adult, severe/critically ill patients	A randomized, open-label, controlled trial	Adalimumab vs. Standard medical care	TNF-α inhibitor	Infections; fever; anemia, neutropenia, lymphopenia etc.
NCT04324021 (70)	Adult (30–79 years), severe/critically ill patients	An open label, controlled, parallel group, 3-arm, multicenter study	Emapalumab vs. Anakinra vs. Standard medical care	IFN-γ inhibitor	Serious infections; skin rash; fever; anemia; coagulopathy etc.
NCT04358614	Adult, moderate patients	A phase 2/3, open label, clinical trial	Baricitinib vs. Standard medical care	JAK1/JAK2 inhibitor	Infections; malignancy; thrombosis: DVT, PE; bleeding; myelofibrosis; anemia, neutropenia, lymphopenia, thrombocytosis, liver enzyme elevations etc.
NCT04321993	Adult, severe/critically ill patients	An open label, non-randomized, parallel group study	Baricitinib vs. Standard medical care		
NCT04320277	Adult (18–85 years), mild/moderate patients	An open label, non-randomized, crossover assignment study	Baricitinib vs. Standard medical care		
NCT04346147	Adult, non-severe patients	A prospective, phase II, randomized, open-label, parallel group study	Baricitinib vs. Hidroxicloroquine vs. Lopinavir/ritonavir vs. Imatinib		
NCT04340232	Adult (18–89 years), without invasive oxygen supplementation	A single arm, open label study	Baricitinib		
NCT04321993	Adult, severe/critically ill patients	A parallel, open-label, non-randomized intervention trial	Baricitinib vs. Standard medical care		
NCT04348695	Adult, severe patients	A randomized, open label, phase II trial	Ruxolitinib plus simvastatin vs. Standard medical care		
NCT04331665	12 Years and older, require supplemental oxygen	A single arm open-label clinical study	Ruxolitinib		
NCT04337359	6–90 years, severe/critically ill patients	A single arm open-label, intermediate-size population	Ruxolitinib		
NCT04338958	Adult, severe/critically ill patients	A single arm, non-randomized open phase II trial	Ruxolitinib		
NCT04348071	Adult (18–89 years), requires supportive care	An adaptive phase 2/3 clinical trial	Ruxolitinib vs. Standard medical care		
NCT04332042	Adult (18–65 years), hospital admission from less than 24 h	A prospective, single cohort, open study	Tofacitinib	JAK1/JAK3 inhibitor	
NCT04322682	40 Years and older, possess at least one high-risk criteria	A randomized, double-blind, placebo-controlled, multi-center study	Colchicine vs. Placebo oral tablet	Inhibition of pyrin and NLRP3 inflammasome activation	Diarrhea; pancytopenia nausea; abdominal pain etc.
NCT04322565	Adult (18–100 years), severe patients	A prospective, phase II, randomized, open-label, Parallel Group Study	Colchicine vs. Standard of care		
NCT04328480	Adult, severe/critically ill patients	A simple pragmatic randomized open controlled trial	Colchicine vs. Local standard of care		
NCT04326790 (71)	Adult, severe/critically ill patients	An open label, randomized, parallel group study	Colchicine vs. Standard of care		
NCT04350320	Adult, admitted in the hospital in the previous 48 hours, with clinical status 3, 4, or 5 of WHO classification.	A phase III, prospective, pragmatic, randomized, controlled and open-label trial	Colchicine vs. Standard of care		
ChiCTR2000029386	Adult, severe/critically ill patients	A prospective, phase II, randomized, open-label, Parallel Group Study	Methylprednisolone vs. Standard of care	Promote the inhibition of HAT and recruitment of HDAC2 activity to downregulate inflammatory genes	Serious Infections: pneumonia, herpes zoster, urinary tract infection; fever; allergy; thrombosis; abnormal blood glucose and pressure; arrhythmia etc.
ChiCTR2000029656	Adult, severe/critically ill patients	A randomized, open-label study	Methylprednisolone vs. Standard of care		
NCT04263402	Adult, severe/critically ill patients	An open, prospective/retrospective, randomized controlled Cohort Study	Methylprednisolone vs. Standard of care		
ChiCTR2000030503	Adult, severe/critically ill patients in ICU	A prospective cohort stud	Blood purification therapy	Remove elevated inflammatory mediators and cytokines	Allergies; fever, thrombosis; hypotension; thrombocytosis; bleeding; air embolism etc.
ChiCTR2000029606	Adult, critically ill patients	An open, randomized controlled trial	Menstrual Blood-derived Stem Cells	Inhibit abnormal activation of T cells and macrophages and induce their differentiation into regulatory T cells and anti-inflammatory macrophages; obstruct the secretion of pro-inflammatory cytokines.	Allergies; fever; arrhythmia etc.
ChiCTR2000031139	Adult (18–80 years), severe/critically ill patients	An open label, single arm study	Embryonic MSCs		
ChiCTR2000030088	Adult (18–80 years), severe/critically ill patients	An open label, randomized, parallel group study	Wharton’s Jelly MSCs		
NCT04269525	Adult (18–80 years), severe/critically ill patients	An open label, single arm study	Umbilical cord-derived MSCs		
NCT04252118	Adult (18–70 years), severe/critically ill patients	An open label, non-randomized, parallel group study	MSCs vs. Standard of care		
NCT04288102	Adult (18–75 years), severe/critically ill patients	A phase II, multicenter, randomized, double-blind, placebo-controlled Trial	MSCs vs. Standard of care		
NCT04276987	Adult (18–75 years), severe/critically ill patients	An open label, single arm, pilot clinical study	Allogenic adipose MSCs		
NCT04273646	Adult (18–65 years), severe/critically ill patients	An open label, randomized, parallel group study	Umbilical cord-derived MSCs vs. Standard of care		

### Inhibition of Cytokines

#### Blocking of IL-6/IL-6R

One meta-analysis of mean IL-6 concentrations demonstrated 2.9-fold higher levels in patients with complicated COVID-19 compared with those with non-complicated disease (74), and another meta-analysis also reported the relation between IL-6 levels and severe condition (75), indicating that IL-6 was a good indicator of poor prognosis in COVID-19 (74). At present, blockers of IL-6/IL-6R have been preliminarily applied in a series of ongoing clinical trials of COVID-19 and further multi-center clinical trials are being carried out (**Table 2**). Tocilizumab (a kind of IL-6 receptor blocker, IL-6R), first approved for rheumatic conditions, can effectively reverse iatrogenic cytokine storms which are caused by CAR-T therapy in patients with hematological malignancies (11, 76). One clinical trial from China (ChiCTR2000029765) reported that tocilizumab made rapid improvements in fever control and respiratory functions in 21 severe patients with COVID-19, and all participants including two seriously ill patients recovered and were discharged from the hospital (77, 78). Roumier et al. reported their experience regarding tocilizumab in 30 COVID-19 patients, suggesting that tocilizumab significantly reduced mechanical ventilation requirements (odd ratio, OR=0.42) and risk of subsequent ICU admissions (OR=0.17) (79). Toniati’s group published a single center study of 100 COVID-19 patients in Italy and also demonstrated that the response to tocilizumab was rapid, sustained, and associated with significant clinical improvements (80). A systematic review and meta-analysis of observational studies found decreased mortality in COVID-19 patients treated with tocilizumab (81). In addition, IL-6 blockade with tocilizumab does not impair the viral specific antibody responses despite of a delayed viral clearance driven by a higher initial viral load, indicating the safety of tocilizumab in patients with COVID-19 (82). Sarilumab is another kind of IL-6R blocker that is being investigated in SARS-CoV-2 infection (83, 84), which may reflect a possible clinical benefit regarding early intervention with IL-6-modulatory therapies for COVID-19 (85). Although not yet approved by Food and Drug Administration (FDA), clazakizumab (a monoclonal antibody against human IL-6) may be helpful in inhibiting the cytokine storms, and related clinical trials are underway worldwide (86). It is also reported that patients with rapidly progressing COVID-19 respiratory failure requiring ventilatory support may benefit from treatment with siltuximab (IL-6 monoclonal antibody) because of reduced mortality and cytokine-driven hyperinflammation (NCT04322188) (66).

#### Blocking of IL-1 Family

There are three important cytokines of IL-1 family in cytokine storms: IL-1β, IL-18, and IL-33, among which the block of IL-1β to counteract the cytokine storms is of great concern (9, 29). Anakinra, a kind of IL-1 receptor antagonist that blocks activity of both IL-1α and IL-1β, has been approved by the FDA and the EDA (European Drug Administration) for the treatment of rheumatoid arthritis, systemic-onset juvenile idiopathic arthritis, and familial Mediterranean fever (67, 87, 88). Anakinra can also be used in the treatment of cytokine storms caused by infection, significantly improving the survival rate of severe sepsis (89). Compared with other cytokine blockers, anakinra has shorter half-life; thus, it is safer and more suitable for severe and critically ill patients. A retrospective cohort study (NCT04324021) reported that high doses of intravenous anakinra inhibit systemic inflammation and were associated with progressive improvement in respiratory function in severe patients with COVID-19 (70). Other clinical trials are also under way to evaluate the use of anakinra in COVID-19 (NCT04330638, NCT04341584, NCT04339712 etc.). Canakinumab, a monoclonal antibody selectively targeting IL-1β, is also being investigated in the treatment for COVID-19. It was proved that canakinumab was safe, well tolerated, and associated with a rapid reduction in the systemic inflammatory response and an improvement in cardiac and respiratory function (68, 69). Nevertheless, strengthened evidence for the application of anakinra and canakinumab in COVID-19 are required in further random controlled trials.

#### Other Blockers

There is no clinical evidence nor any registered clinical trials assessing the possibility of IL-18 blockers and IL-33 blockers in COVID-19. TNF-α and IFN-γ are also key inflammatory cytokines and attractive targets in the control of cytokine storms (6, 90, 91), and clinical trials are ongoing to test these blockers in COVID-19 (ChiCTR2000030089, NCT04324021) (70, 92). JAK/STAT (janus kinase/signal transducers and activators of transcription) signal transduction pathway, a common downstream signaling pathway of various cytokines, can block multiple targeted cytokines at the same time if inhibited (93, 94). However, its possible side effects cannot be ignored, such as increased risk for pulmonary embolism (PE), liver enzyme elevations, hematological abnormalities and suppression of antiviral immunity (94–96). Several representative JAK inhibitors, such as tofacitinib, baricitinib, and ruxolitinib, are currently being investigated to determine whether they can be applied to the treatment of COVID-19 (NCT04332042, NCT04348695, NCT04321993, NCT04348695, etc.) (94). Baricitinib and ruxolitinib are selective inhibitors of JAK1/JAK2 which is responsible for multiple cellular signals including the proinflammatory IL-6 and works as immunomodulator decreasing the cytotoxic T lymphocytes and increasing the regulatory T cells (97, 98). A phase 2/3 clinical trial (NCT04358614) showed that all clinical characteristics and respiratory function parameters significantly improved in the baricitinib-treated group compared to the baseline, and no serious infections, cardiovascular and hematologic adverse effects occurred after treatment (29). Although tofacitinib is a selective inhibitor of JAK1/JAK3, it is suggested that for patients not on tofacitinib that this be initiated, but rather for those already on it that it can potentially be continued during a pandemic (99). Colchicine can inhibit the inflammasome activation of pyrin and NLRP3 (NLR Family Pyrin Domain Containing 3), and are also currently under way to be evaluated in the treatment of COVID-19 (71, 100, 101). A single-center cohort study showed that patients treated with colchicine had a better survival rate as compared with standard care at 21 days of follow-up, and the adverse effects were similar for two groups, which may support the rationale of use of colchicine for the treatment of COVID-19 (100). Another randomized clinical trial of 105 COVID-19 patients also suggested that low-dose of colchicine combined anti-inflammatory action with a favorable safety profile (71). Moreover, it is also recommended as a therapeutic option in patients who have contraindications to other drugs or in the context of shortage/unavailability of anti-viral drugs (such as in underdeveloped countries) due to high availability (101).

### Corticosteroids

Corticosteroids, a type of steroid hormones, exhibit anti-inflammatory activity *via* binding to the cytoplasmic corticosteroid receptor, which leads to inhibitions of HAT (histone acetyltransferase) and recruitment of HDAC2 (histone deacetylases 2) activity to downregulate inflammatory genes (102). Thus, corticosteroids have been widely used to control cytokine storms. Data from a limited-size trial showed that the early use of low or medium doses of methylprednisolone had a positive effect for patients with severe COVID-19 (103). A single-blind, randomized, controlled trial in Iran (trial identifier: IRCT20200404046947N1) suggested that methylprednisolone pulse could be an efficient therapeutic agent for hospitalized severe COVID-19 patients at the pulmonary phase (104). Although there was no significant difference in mortality, patients receiving methylprednisolone treatment seemed to be with a faster improvement of oxygen saturation, decrease in CRP and IL-6 level, and less demand for invasive ventilation (104, 105). However, adverse effects (including serious infection and edema etc.) were also observed in several patients after the methylprednisolone treatment (103–105). In addition, there are heated debates as to whether corticosteroid therapy will delay viral clearance in COVID-19 (106, 107). Therefore, the pros and cons should be carefully weighed before using glucocorticoids in the treatment of COVID-19 patients. It is very important for clinicians to master the time and dose of corticosteroids for the treatment of severe patients, especially before the infection occurs. In addition, great caution should be exercised for patients who already have hypoxemia for various reasons or those who take glucocorticoids regularly due to other chronic diseases.

### Blood Purification Therapy

It has been demonstrated that the blood purification system, such as plasma exchange, adsorption, perfusion, blood/plasma filtration, can remove inflammatory factors and then reduce tissue damage of hyper-activated inflammatory responses (108). Li et al. demonstrated that the artificial liver blood purification system could rapidly remove inflammatory mediators, block cytokine storms, favor the balance of fluid, electrolytes and acid-base, and thus improve treatment efficacy in critical illnesses (109, 110). Ma et al. reported that three COVID-19 patients who received blood purification therapy were tolerable and effective in limited experiences (111). Successful recovery of a severe patient was also presented in one case report (112). Yang’s group developed a blood purification protocol for patients with severe COVID-19 based on previous experience in SARS and MERS, including four major steps: (i) to assess whether patients with severe COVID-19 require blood purification; (ii) to prescribe a blood purification treatment for patients with COVID-19; (iii) to monitor and adjust parameters of blood purification; (iv) to evaluate the timing of discontinuation of blood purification (113). Last but not least, possible adverse effects in blood purification therapy (such as allergies, thrombocytosis, bleeding, air embolism etc.) should be timely identified and controlled to ensure safe and effective treatment (109–113).

### Stem Cell Therapy

As an important member of the stem cell family, mesenchymal stem cells (MSCs) not only have potentials for self-renewal and multidirectional differentiation but also have strong anti-inflammatory and immune regulatory functions (114–117). In addition, MSCs inhibit abnormal activation of T lymphocyte cells and macrophages and induce their differentiation into regulatory T cells and anti-inflammatory macrophages (117). MSCs also obstruct the secretion of pro-inflammatory cytokines, thereby reducing the occurrence of cytokine storms (114, 115, 117, 118). Currently, over thirty clinical trials about intravenous administration of MSCs in COVID-19 patients have been officially registered (www.clinicaltrials.gov), but most of them are under the recruitment phase. Recently, Zhao et al. reported the results of seven severe and critically ill patients with COVID-19 receiving MSCs transplantation therapy, which showed improved prognosis and effective avoidance of cytokine storms with no obvious side effects (119). Furthermore, other stem cell therapies have been initiated in clinical trials, such as human menstrual blood-derived stem cells (ChiCTR2000029606) and embryonic stem cells (ChiCTR2000031139).

## Concluding Remarks

COVID-19 has been listed as an international public health emergency by WHO and the treatment of severe and critically ill patients is the burning issue in current prevention and control. As cytokine storm is one of the most common causes of mortality in COVID-19, therapeutic approaches to manage cytokine storm may provide a novel avenue to decrease the COVID-19 associated morbidity and mortality. Hyperactivated innate immune responses, dysregulation of ACE2 expression and its downstream pathway may be possible mechanisms. In the treatment of COVID-19, high levels of attention must be paid to the identification of the occurrence cytokine storm. Tailored immunoregulatory therapies to control and resist the progress of cytokine storms in early stages of COVID-19 can greatly improve prognosis and reduce mortality rates. Although several therapies used to control cytokine storm have entered the stage of clinical trials for the treatment of COVID-19, the limited source and potential adverse effects have delayed widespread application in clinical treatment. Moreover, the best timing of anti-cytokine storm therapies remains to be explored, as well as novel therapeutic methods which are more effective and tolerated.

## Author Contributions

LT, YH, and HM: writing, original draft preparation. LT: table and figure preparation and editing. ZY, YH, and HM: review and editing. All authors contributed to the article and approved the submitted version.

## Funding

This research was supported by funds from the Key Special Project of Ministry of Science and Technology of China (no. 2020YFC0845700), Scientific Research Projects of Chinese Academy of Engineering of China no. 2020-XY-70 (2020-KYGG-01-07), and Fundamental Research Funds for the Central Universities of China (no. 2020kfyXGYJ029).

## Conflict of Interest

The authors declare that the research was conducted in the absence of any commercial or financial relationships that could be construed as a potential conflict of interest.
